# Nanocellulose Filled Bio-Based PVA/Chitosan Nanocomposites: Structure–Property Relationships Toward Advanced Food Packaging Films

**DOI:** 10.3390/polym17233122

**Published:** 2025-11-24

**Authors:** Konstantinos Papapetros, Georgios N. Mathioudakis, Dionysios Vroulias, Nikolaos Koutroumanis, Amaia Soto Beobide, Olympia Kotrotsiou, Giannis Penloglou, Konstantinos S. Andrikopoulos, George A. Voyiatzis

**Affiliations:** 1 Foundation for Research and Technology—Hellas (FORTH), Institute of Chemical Engineering Science (ICE-HT), Stadiou St., GR 265 04 Patras, Greece; 2 Department of Chemical Engineering, University of Patras, GR 265 04 Patras, Greece; 3 Application Driven Research & Innovative Engineering (ADRINE), Patras Science Park, Stadiou Street, Platani, GR 265 04 Patras, Greece; 4 Centre for Research and Technology Hellas (CERTH), Chemical Process and Energy Resources Institute (CPERI), 6th km. Charilaou-Thermi Rd, Thermi, GR 570 01 Thessaloniki, Greece; 5 Department of Physics, University of Patras, GR 265 04 Patras, Greece

**Keywords:** PVA, chitosan, nanocellulose, lignocellulose, nanocomposites, biodegradable food packaging

## Abstract

Biodegradable chitosan/poly(vinyl alcohol) (PVA) composite films were reinforced either with nanocrystalline cellulose (CNC) or nano-lignocellulose (NLC) and evaluated across a polyparametric design of five matrix ratios and three filler levels for active food packaging applications. ATR-FTIR, DSC, XRD, and SEM demonstrated that 1–5% nanocellulose loading induced a single relaxation temperature (T_g_), homogenized the morphology, and enhanced the crystallinity of blend material, evidencing improved thermodynamic compatibility. SEM confirmed uniform filler dispersion up to 5% loading in PVA-rich matrices, whereas limited aggregation appeared in chitosan-dominant systems. CO_2_ barrier property (CO_2_ permeability coefficients) was diminished by more than two orders of magnitude and fell below 0.01 Barrer in CNC-filled 25-75 and NLC-filled 75-25 blends, while permeability to O_2_ and N_2_ remained undetectable under identical conditions. Meanwhile, Young’s modulus increased to 3.9 GPa, and tensile strengths of up to 109 MPa were achieved, without affecting the ductility in specific loading values. These data confirm that tailored selection of the filler/matrix combination, rather than elevated nanocellulose content, can simultaneously optimize barrier performance and mechanical integrity. The study therefore offers a scalable, water-based route for producing optically transparent nanocomposite membranes that satisfy either strict modified atmosphere or/and rigid packaging applications and advance the transition toward compostable/or even edible high-performance food contact materials.

## 1. Introduction

Driven by the increasing demand for sustainable, biodegradable materials, biopolymer-based composites have been extensively investigated as alternatives to petroleum-derived polymers. Various biopolymers have been used as alternatives, focusing on polyhydroxyalkanoates (PHAs), polylactic acid (PLA) and polysaccharide polymers, such as starch, cellulose, and chitosan [1,2,3,4]. Among these, chitosan, a linear polysaccharide of β-(1→4)-linked D-glucosamine and N-acetyl-D-glucosamine units—derived from chitin—with key functional groups, including primary amino (NH_2_) and hydroxyl (OH) groups that facilitate intermolecular interactions like hydrogen bonding and electrostatic interactions, has emerged as a promising candidate due to its biocompatibility, biodegradability, and inherent antimicrobial properties [5,6]. Its properties are dictated by its structure, particularly its degree of deacetylation, molecular weight, and the pattern of acetylation, which influence factors, like solubility, charge density, and crystallinity. However, its practical application is often limited by drawbacks, such as poor mechanical strength, thermal instability, and pronounced hydrophilicity, which compromises its performance in humid environments [7,8]. Moreover, considerably high rigidity, along with elevated water vapor and oxygen permeabilities, has been observed in chitosan films prepared by film casting [4,9]. Thus, further optimization of both the mechanical and barrier properties of these biopolymer membranes is highly required. To optimally meet application needs, combining different biopolymers to create composite materials is an important means of achieving improved properties and/or functionality. The hypothesis that molecular interactions between chitosan and another biodegradable polymer with complementary properties, if properly realized, can lead to materials with additional properties than those of pristine materials was recently realized with gelatin [10].

In the present work, in order to optimize the chitosan properties, two different approaches are used: the introduction of compatible nanomaterial inclusions and/or blending with a water-soluble polymer (even synthetic)—which do not affect the environmental footprint. To address these limitations, blending chitosan with synthetic polymers, like polyvinyl alcohol (PVA), has gained traction, as PVA enhances mechanical robustness, flexibility, and water resistance while retaining the eco-friendly profile of the composite material. The use of these blends is not only focused on packaging applications but also on biomedical ones, such as wound healing, controlled drug delivery, and others [11,12,13]. Nevertheless, since optimal interfacial compatibility between chitosan and PVA remains debatable and depends on parameters such as the molecular weight of PVA and chitosan’s degree of deacetylation, it has become a necessity to develop novel strategies to further improve the blend material’s overall properties [14,15,16].

The addition of nanocellulose, either as nanocrystalline cellulose (CNC) or nano-lignocellulose (NLC), is used as a reinforcing material in blend systems due to its large surface area per mass unit that allows the formation of extensive hydrogen bonds with hydrophilic polymeric matrices. Notably, nanocellulose enhances the interfacial stress transfer of PVA [17], elasticity, biocompatibility, and gas barrier properties in both PVA [18,19,20] and chitosan composites [21,22]. These studies have primarily focused on the incorporation of plant-derived nanocellulose into selected PVA/chitosan blend ratios, with their effects on material properties for packaging applications being examined. PVA was also utilized as the primary matrix, with chitosan being incorporated at varying concentrations (e.g., 10 wt% [23], 25 wt% [24], 30 wt% [25], and 5–20 wt% [26]), while nanocellulose was added at 1% or 3 wt% However, the utilization of chitosan as the primary matrix, along with critical aspects such as gas permeability measurements (CO_2_, O_2_, and N_2_) and mechanical performance—parameters crucial for packaging applications—has not been systematically explored. In a related study by Solikhin et al. [27], the addition of 15 wt% chitosan and varying cellulose CNC loadings to PVA was reported to yield improved miscibility, and enhancements in mechanical performance were observed with the incorporation of minimal CNC amounts (0.5%).

While considerable attention has been directed toward chitosan/PVA composites reinforced with nanocellulose for packaging applications, prior studies have largely adopted fixed blend ratios or a single type of cellulosic reinforcement. In such studies, either the chitosan/PVA ratio or the nanocellulose content was held constant, restricting the analysis of compositional variations on overall performance. Additionally, the scope of characterization has typically been narrowed, with mechanical properties examined only sporadically and gas permeability, a critical parameter for packaging applications, remaining unaddressed. To date, no study has systematically explored the combined impact of modifying both the chitosan/PVA matrix composition and the type/loading of nanocellulosic reinforcements on a comprehensive set of functional properties and the possible selective effect of each inclusion on the properties of PVA-rich or chitosan-rich blends. By addressing these limitations, the current study introduces a novel approach by integrating two structurally distinct nanocelluloses (nanocrystalline cellulose, CNC, and nano-lignocellulose, NLC) into chitosan/PVA matrices with systematically varied ratios. A thorough assessment of gas permeability behavior (CO_2_, O_2_, N_2_), mechanical performance, and blend miscibility is conducted, elucidating structure–property relationships that advance the development of biopolymer nanocomposites for sustainable packaging solutions. This multiparametric investigation not only bridges existing knowledge gaps but also establishes a methodological framework for tailoring material performance through synergistic adjustments in polymer composition and nanoscale reinforcement strategies. The novelty of this study lies in its ability to tailor chitosan/PVA/nanocellulose composite membranes to meet specific application demands, such as high ductility for modified atmosphere packaging (MAP) or increased modulus of elasticity for rigid packaging, by optimizing both the blend ratio and nanocellulose loading, a level of customization not previously addressed in prior work that either fixed the blend ratio or filler content.

## 2. Materials and Methods

### 2.1. Materials

Chitosan (190 to 310 kDa, CAS Number: 9012-76-4) glacial acetic acid (min 99.8%, CAS Number: 64-19-7) and polyvinyl alcohol (PVA) (Mw of 89−98 kDa with 99+% hydrolyzed, CAS Number: 9002-89-5) were purchased from Sigma-Aldrich (St. Louis, MO, USA). Aqueous 11.5–12.5 wt% CNC suspension (CAS Number: 9004-34-6) was purchased from the University of Maine (Orono, ME, USA). Nano-lignocellulose (NLC) was produced and kindly provided by CERTH (Thessaloniki, Greece). Specifically, hardwood biomass underwent thermal treatment at 190 °C for 90 min and was subsequently milled/grinded in 500 mL stainless steel bowls using a laboratory planetary ball mill (Pulverisette 5 Planetary Mill, premium line, Fritsch GmbH, Idar-Oberstein, Germany), modifying an established protocol for producing various grades of nanocellulose [28]. The pretreated suspension was placed in the milling bowls together with stainless steel balls of 15 mm and 3 mm diameter, maintaining a respective mass ratio of 0.74 g g^−1^. The total ball-to-material (biomass) mass ratio was set to 5.6 g g^−1^. Milling was carried out at rotation speed of 300 rpm for a total of 8 h, using 1 min on/2 min off cycles prevent overheating, with reversed rotation applied in every other cycle. Finally, ultrapure water was obtained by a MilliQ (Millipore, MA, USA) apparatus water purification unit.

### 2.2. Sample Preparation

#### 2.2.1. Chitosan–Cellulose Composites

About 1 wt% chitosan powder was dissolved in 1% *v*/*v* glacial acetic acid aqueous solution under mechanical stirring at room temperature for 3 h. The final solution was allowed to rest overnight until the insoluble moieties precipitated, and then the supernatant clear pale-yellow solution was collected. About 20 mL aqueous suspension containing different nanocellulose concentrations (0, 1, 5, 10 wt%) of CNC or NLC was mixed with the chitosan solution. The chitosan/nanocellulose mixtures were homogenized under vigorous mechanical stirring for 2 h at room temperature (1000–1200 rpm depending on the viscosity of the solution) before being sonicated for 10 min at 45 W. The final homogenized mixtures in the form of stable suspensions obtained were cast on glass petri dishes and dried for 1 week at room temperature, without further treatment before characterization.

#### 2.2.2. Chitosan–PVA–Cellulose Composites

Aqueous solutions of 4 wt% PVA were prepared by dissolving the polymer in triple-distilled water at 85 °C under (magnetic) stirring for 1 h. After stirring, the transparent PVA solution was left at room temperature to cool down. Several mixtures of chitosan/PVA blends at weight ratios of 100-0, 75-25, 50-50, 25-75, and 0-100 were prepared, with the processes of adding cellulosic suspensions and film casting being the same as described above. In this set of experiments, the chitosan used was pristine (unmodified), serving as the polymer matrix into which the nanocellulose was incorporated. All the prepared samples are summarized in Table 1, which were used for the following characterization and analysis.

### 2.3. Analytical Techniques

#### 2.3.1. Scanning Electron Microscopy (SEM)

Scanning Electron Microscopy (SEM) was employed to evaluate the morphological characteristics (including size and shape) of cellulosic material and its (homogeneous) distribution within chitosan and chitosan/PVA blend matrices. To identify structural variations resulting from increased filler content, as well as potential defects or agglomerates, film samples were cryogenically fractured using liquid nitrogen. Imaging was performed with a Zeiss SUPRA 35VP SEM system (Carl Zeiss AG, Oberkochen, Germany) at an accelerating voltage of 2–5 kV, optimized for individual sample stability.

#### 2.3.2. Attenuated Total Reflection Fourier Transform Infrared Spectroscopy (ATR-FTIR)

ATR-FTIR spectra of solid samples were acquired using a Bruker Alpha-II Diamond ATR spectrometer (Bruker Optics GmbH, Ettlinger, Germany), covering the wavenumber range of 400–4000 cm^−1^ (a zoomed-in spectral window was presented to highlight the most relevant regions where compositional or interaction-related variations occurred). Spectra were recorded by co-adding 24 scans at a resolution of 4 cm^−1^, with a minimum of five replicate measurements averaged for statistical reliability.

#### 2.3.3. X-Ray Diffraction (XRD)

X-ray diffraction was used to identify the crystalline phase and detect changes in chitosan/PVA blends before and after adding various loadings of nanocellulose materials. XRD measurements were carried out with a Bruker D8 Advance diffractometer equipped with a Cu lamp (λ_CuKa_ =1.54046 Å) at a scanning rate 0.5°/min over a range 5–30° (2θ).

#### 2.3.4. Differential Scanning Calorimetry (DSC)

Differential scanning calorimetry (DSC) measurements were performed on a Q100 system (TA Instruments, New Castle, DE, USA) equipped with a liquid nitrogen cooling accessory. To investigate the melting behavior of PVA in each chitosan/PVA blend and in their nanocomposites, each sample (approximately 6–8 mg) was heated from room temperature to 240 °C at a rate of 10 °C min^−1^ under a nitrogen flow of 50 mL min^−1^. Data were collected from the first heating cycle to reflect the actual state of the prepared composites. The Crystallinity Index (Cr.I %) of PVA was determined from the endothermic melt peak using

(1) $$Cr.I(\%)=\frac{{\Delta H}_{m}}{{\Delta H}_{m}^{0}(1-\varphi )}\times 100$$

where ${\Delta H}_{m}$ is the measured heat fusion, ${\Delta H}_{m}^{0}$ is the standard heat fusion for 100% crystalline PVA (138.6 J g^−1^) [29], and φ is the weight fraction of both the added chitosan and fillers.

#### 2.3.5. Gas Permeability Measurements

Single-gas permeation was evaluated using the Wicke–Kallenbach method. A circular membrane (effective area: 6.97 cm^2^) was installed in a custom permeation cell, with the retentate and permeate chambers sealed by Viton O-rings (Chemours Company, Wilmington, DE, USA) and held under vacuum clamp. The cell temperature was maintained at 30 °C via two coil heaters (BL Sistemi, Roma, Italy), and a thermocouple (RS Components, Corby, UK) was controlled by a dedicated temperature regulator. Gas flow rates on both sides were set with two mass flow controllers (Aera FC7700C, Hitachi Metals, Ltd., now Proterial, Ltd., Toyosu, Koto-ku, Tokyo, Japan) at atmospheric pressure. Helium served as the sweep gas at 20 cm^3^ min^−1^. Permeate compositions were determined on a Shimadzu gas chromatograph (GC-2014, Shimadzu corporation, Kyoto, Japan) fitted with a thermal conductivity detector and Porapak Q column (Agilent Technologies, Santa Clara, CA, USA) for CO_2_ analysis, while N_2_ and O_2_ were measured using a quadrupole mass spectrometer with a secondary electron multiplier. The O_2_ signal at *m*/*z* = 32 was calibrated against known O_2_ standards.

#### 2.3.6. Mechanical Characterization

A hydraulic mechanical testing system (MTS R58 Mini Bionix, MTS Systems Corporation, Eden Prairie, MN, USA) was employed for the tensile tests of both neat blend and nanocomposite materials. The MTS system was equipped with a load cell of 25 kN. Tensile properties of all membranes were determined according to modified ASTM D882 (~5 cm length and ~5 mm width) [30], while the crosshead moving speed was set to 3 mm min^−1^.

## 3. Results

### 3.1. Effect of Chitosan/PVA Ratio on the Blends’ Properties

**Figure 1:**
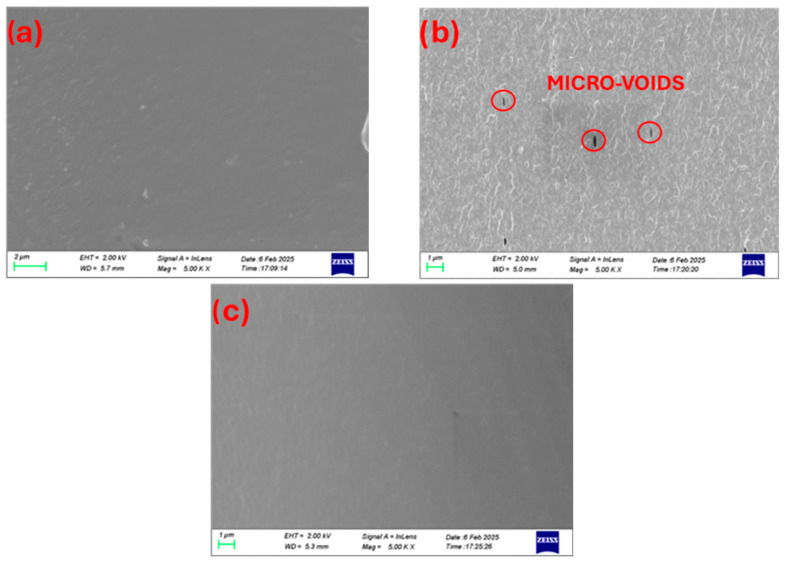
Cross-section surface SEM images of chitosan/PVA blends at high magnification (5.00 Kx). (**a**) 25-75, (**b**) 50-50, and (**c**) 75-25.

The miscibility of the chitosan/PVA blend was observed to be significantly influenced by the relative proportions of the two polymers, as evidenced by SEM analysis of cryo-fractured cross-sectional morphologies. In the 25-75 chitosan/PVA formulation (Figure 1a), a uniform and continuous structure was noted, signifying effective compatibility and homogeneous distribution of chitosan within the PVA matrix. By contrast, the 50-50 blend (Figure 1b) displayed a markedly rougher surface morphology characterized by prominent micro-voids indicative of phase separation and weak interfacial adhesion between the polymer phases at this composition [14]. In the 75-25 chitosan/PVA blend (Figure 1c), a smooth and continuous morphology was again observed, suggesting enhanced miscibility when chitosan predominates in the matrix. For comparison, the morphologies of pure chitosan (100-0) Figure S2a and pure PVA (0-100) are shown in Figure S3j. [17]. Moreover, according to the Flory–Huggins theory, symmetric blending ratios may be favorable to intramolecular interactions within each polymer chains, reducing the blend miscibility. These findings imply that intermediate ratios may induce partial immiscibility, potentially due to disruptions in hydrogen bonding equilibrium or reduced molecular entanglement. Chain entanglements act as physical crosslinks that contribute to the mechanical integrity and phase stability of polymer blends [31]. A lower degree of entanglement between polymer chains can weaken interfacial adhesion and promote phase separation.

Differential scanning calorimetry (DSC) was utilized to investigate the thermal transitions of chitosan/PVA blends and evaluate their miscibility by monitoring changes in glass transition temperature (T_g_) and melting point (T_m_) (Table 2). While identification of PVA’s glass transition is highly influenced by the residual water content, the molecular weight, and sample pretreatment (e.g., drying), in the present study, the T_g_ was well defined and found at ~45 °C, while chitosan showed a glass transition at ~90 °C. It should be noted, however, that chitosan’s glass transition is a subject of controversy, with the source of extraction or the method followed strongly influencing it [32].

**Figure 2:**
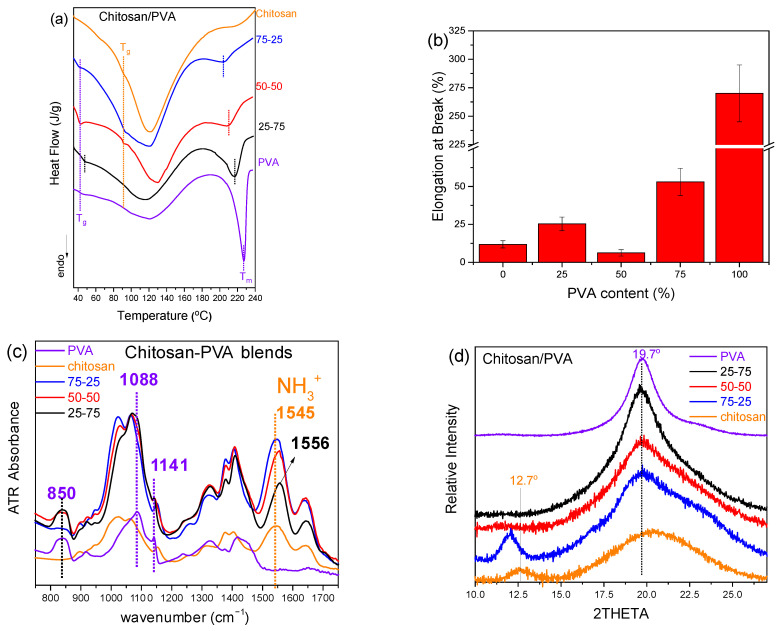
Comparative analysis of various chitosan/PVA blend ratios by (**a**) DSC, (**b**) elongation at break vs. PVA content bar plots, (**c**) ATR-FTIR spectra, and (**d**) XRD graphs.

As illustrated in Figure 2a, neat chitosan and PVA were found to display distinct T_g_ and T_m_ values, respectively. In blended samples, the detection of a single or dual T_g_ is a key indicator of polymer compatibility. For the 25-75 chitosan/PVA ratio, a single broadened T_g_ was detected, indicative of miscibility and the development of a single-phase region characterized by overlapping polymer segment dynamics. Conversely, the 75-25 and 50-50 blends exhibited two distinct T_g_ values, signifying phase separation and the coexistence of immiscible domains [14,15,33]. Furthermore, a gradual decrease in T_m_ with higher PVA content was noted, reflecting structural reorganization within PVA’s crystalline regions [34].

The relationship between miscibility and mechanical performance was further investigated through elongation at break values (Figure 2c), as evidenced by the corresponding stress–strain curves (Figure S1). An almost steady increase in elongation was observed with rising PVA content, peaking at 100% PVA. This behavior reflects the flexibility of PVA and its capacity to improve the ductility of the blend system. Interestingly, the lowest elongation values were recorded for the 50-50 composition, aligning with DSC (Figure 2a) and SEM (Figure 2b) findings that identified phase separation at this blend ratio [35]. The diminished elongation at this intermediate ratio was attributed to weak interfacial adhesion and inefficient stress transfer between the polymer phases, as are characteristic of immiscible systems.

Finally, ATR-FTIR and XRD were also used to examine the behavior of the prepared blends related to the mixing ratio (Figure 2c and Figure 2d, respectively). Due to similar spectral characteristics in both PVA and chitosan FTIR spectra (800–1700 cm^−1^), few peaks are discrete, especially those of protonated chitosan (protonated amine), with NH_3_^+^ str. vibration at 1545 cm^−1^ being notably shifted to 1556 cm^−1^ in the case of the 25-75 blend, indicating probable interaction between the chitosan and PVA, which strongly depended on composition. However, the PVA characteristic peaks of O-C-C str. (1141 cm^−1^) and C-OH str. (1088 cm^−1^) vibrations remained unaffected. The characteristic XRD peak of chitosan at approximately 12° (12.7°), which is attributed to regions of partial crystalline organization due to intra- and/or intermolecular hydrogen bonding (between NH_3_^+^ and –OH groups) [36], exhibited notable variations in intensity or even in its detection depending on the composition of the chitosan/PVA blend. The contribution of the well-defined crystalline structure of PVA is observed through the diffraction peak of the (101) plane at 19.7°. In the 75-25 chitosan/PVA blend, the intensity of the chitosan-associated peak at ~12° was higher than that of neat chitosan, suggesting that the addition of a small amount of PVA promotes intermolecular interactions between chitosan and PVA chains. These interactions, mainly hydrogen bonds between hydroxyl and amino groups, can restrict chain mobility and lead to a higher degree of local ordering within the chitosan phase. To further explain these observations, we suggest that the slight shift of the ~12° diffraction peak to lower 2θ in the 75-25 blend (i.e., an increase in d-spacing) is attributed either to altered chain packing caused by hydrogen bonding with PVA and/or to the presence of (bound) water of PVA that may potentially lead to swelling of the chitosan crystal lattice, while the concurrent increase in peak intensity likely reflects the enhanced local ordering and/or larger crystallite domains of the chitosan-associated structure (possibly aided by reduced amorphous background or preferred orientation effects).

In contrast, in the 50-50 and 25-75 compositions, this peak is completely absent. This can be attributed to two concurrent mechanisms: on the one hand, the increasing PVA content promotes intermolecular interactions that disrupt the structural organization of chitosan, leading to the loss of the characteristic peak, while on the other hand, the reduced chitosan content may not allow a sufficiently distinct contribution to be detected in the diffraction pattern.

These findings confirm that morphological observations, supported by DSC, mechanical property tests, FTIR, and XRD, can act as a reliable indicator of blend miscibility, confirming the immiscibility of the 50-50 blend ratio, while the 75-25 ratio revealed an intermediate miscibility profile.

### 3.2. Effect of Cellulose Type/Loading on Chitosan/PVA Blends’ Properties

#### 3.2.1. Microscopic Properties

##### Morphological Characterization

**Figure 3:**
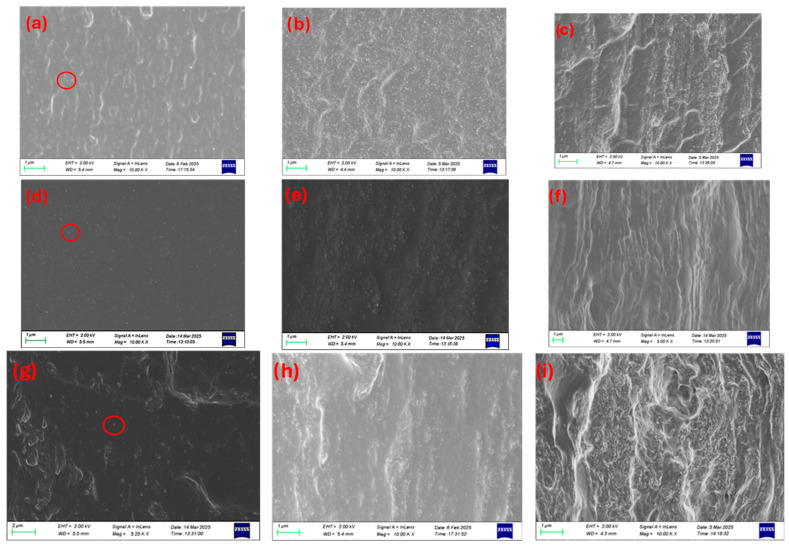
Cross-section surface SEM images of chitosan/PVA/CNC composites at high magnification: (**a**) 25-75 1% (10.00 Kx), (**b**) 25-75 5% (10.00 Kx), (**c**) 25-75 10% (10.00 Kx), (**d**) 50-50 1% (10.00 Kx), (**e**) 50-50 5% (10.00 Kx), (**f**) 50-50 10% (5.00 Kx), (**g**) 75-25 1% (5.25 Kx), (**h**) 75-25 5% (10.00 Kx), (**i**) 75-25 10% (10.00 Kx). The scale bar in all images correspond to 1 μm except for (**g**), which is 2 μm. The circles indicate the presence of CNC.

The cryo-cut SEM images reveal the morphological evolution and dispersion behavior of CNC within chitosan/PVA matrices at different blend ratios and CNC loadings. The needle-shape structures of CNC have a mean width of 40 ± 10 nm and characteristic low length (350 ± 100 nm), giving an aspect ratio of around 10 [17]. In the 25-75 chitosan/PVA composites (Figure 3a–c), CNC dispersion appears relatively homogeneous at 1 and 5% loading (a and b), with minimal agglomeration and a smooth matrix. As the CNC content increases to 10% (c), the surface roughness becomes more pronounced, and visible CNC-rich domains emerge, indicating agglomeration and decreased dispersion uniformity. For the 50-50 blends (Figure 3d–f), the 1% CNC-loaded sample (Figure 3d) exhibits a similarly smooth morphology with good CNC integration, while the 5% loading gives a moderate behavior. The 10% CNC-loaded sample (Figure 3f) displays increased microstructural heterogeneity and CNC clustering, suggesting a critical threshold beyond which dispersion is compromised. In the 75-25 chitosan/PVA composites (Figure 3g–i), even the 1% CNC sample (g) shows signs of CNC aggregation, and these features become more evident in the 5% and 10% (h and i), with clear phase separation and agglomerate formation dominating the fractured surface. A general trend observed across all blend ratios is that CNC dispersion deteriorates with increasing nanofiller content, while higher chitosan content also appears to correlate with reduced CNC dispersion quality (Figure S2). This suggests that PVA-rich matrices may provide a more favorable environment for CNC stabilization and uniform distribution, which is likely due to better compatibility and hydrogen bonding interactions between CNC and PVA.

In contrast to the CNC-based composites, the SEM images of chitosan/PVA membranes incorporated with long fibrous NLC do not exhibit distinct dispersion features within the cross-sectional area. This discrepancy is primarily attributed to the morphological characteristics of NLC, which consists of high aspect ratio fibers typically several microns in length and approximately 50 nm in diameter, as shown in our previous work [17]. During membrane formation, these elongated fibers tend to be settled not in a well-defined orientation, thereby being beneath the cryo-fractured surface. As a result, the distribution and orientation of NLC within the matrix cannot be reliably assessed through visual inspection of cryo-cut cross-sections (Figure S3), similarly to PVA and chitosan composites (Figure S4). Consequently, alternative characterization techniques may be required to evaluate the dispersion state and structural integration of NLC within the composite membranes. An example of well-dispersed inclusions in the scale of microns can be found in optical images of Figure S5, in which CNC composites (Figure S5a) show great transparency, while NLC is well dispersed up to 5% loading in each case (Figure S5b,c).

##### Structure at Molecular Level (ATR-FTIR)

FTIR spectroscopy is a straightforward technique for studying molecular interactions and compatibility in chitosan/PVA/cellulose nanocomposites, as it directly probes the vibrational modes associated with functional groups responsible for hydrogen bonding. Additionally, spectral analysis can highlight changes in polymer backbone conformation and crystallinity induced by nanocellulose incorporation [17]. Thus, ATR-FTIR offers a rapid, non-destructive approach to link the effect of cellulose type/loading from molecular to macroscopic, such as mechanical and gas barrier properties.

**Figure 4:**
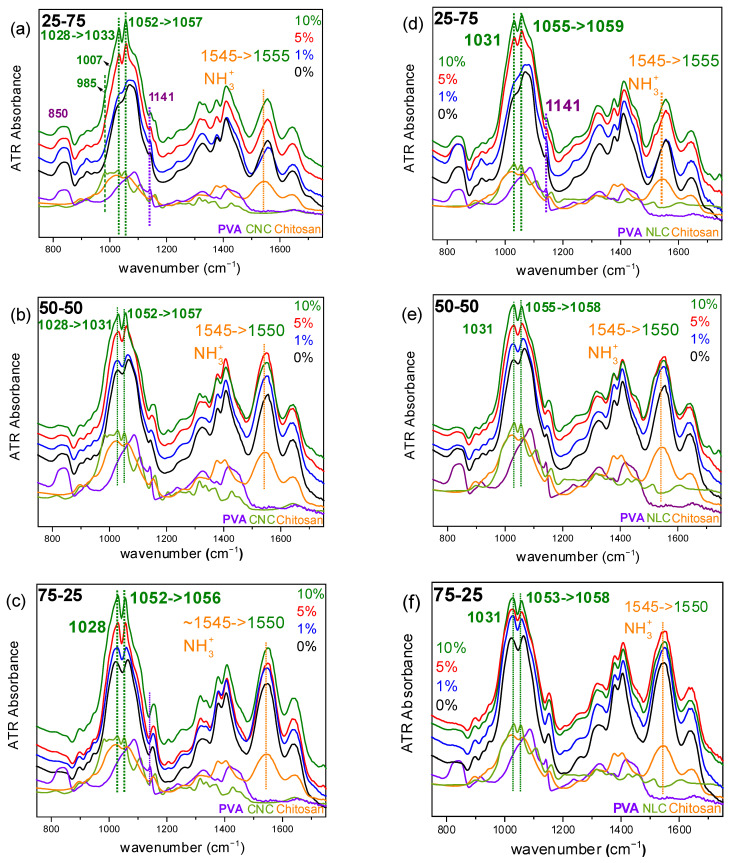
ATR-FTIR spectra in the 700–1700 cm^−1^ spectral window of chitosan/PVA/nanocellulose composites: (**a**) 25-75 x% CNC, (**b**) 50-50 x% CNC, (**c**) 75-25 x% CNC, (**d**) 25-75 x% NLC, (**e**) 50-50 x% NLC, (**f**) 75-25 x% NLC.

As discussed earlier in Figure 2c, there are several peaks in the 800–1700cm^−1^ spectral area of chitosan and PVA, which overlap, while only a few are distinct, with PVA’s CH_2_ bending vibration at 840 cm^−1^ and chitosan’s NH_3_^+^ str. vibration at 1545 cm^−1^ being characteristic. In Figure 4a–c, ATR spectra of CNC composites of the various chitosan/PVA ratio blends are represented, in which the initial frequency of chitosan’s NH_3_^+^ vibration is shifted to higher frequency on each blend by CNC addition. Commonly, peaks observed between 950–1150 cm^−1^ are attributed to C-O stretching vibrations, with shifts in these peaks indicating alternations in the attached hydroxyl group, such as hydrogen bonding. Cellulose, similarly to chitosan and PVA, displays strong peaks in this region due to its extensive structure containing abundant C-O groups.

In a detailed spectroscopic study under controlled temperature and humidity conditions, Maréchal and Chanzy attributed many of these bands to C-O vibrations of the primary alcohol groups (C_6_H_2_–O_6_H at ~1000, 1015, and 1035 cm^−1^) and the two secondary alcohols (specifically C3–O3H at ~1060 cm^−1^ and C2–O2H at ~1110 cm^−1^) within each glucopyranose unit. The three distinct peaks associated with the primary alcohols correspond to different conformations, determined by the orientation of the C6H_2_–O6H group relative to the C5–C6 bond, with the ~1035 cm^−1^ peak being dominant for the crystalline allomorph I_β_ [37].

The vibrational energy of O–H groups, as well as adjacent C–O bonds, is significantly affected by the formation of intra- and intermolecular hydrogen bonds. Variations in the strength of these bonds, due to physical or chemical modifications, can cause peak shifts for both the O–H and adjacent C–O stretching vibrations, typically on the order of ~10 cm^−1^ [38]. It has already been discussed [37] that strengthening hydrogen bonding leads to a decrease in the O–H vibrational frequency, while it has the opposite effect on the associated C–O vibrations.

Indeed, in all CNC composite systems (Figure 4a–c), the characteristic C–O stretching vibrations of cellulose at 1052 cm^−1^ (as well as the one at 1028 cm^−1^ in the 25-75 blend) are observed at higher frequencies within the composite membranes (1033 and 1057 cm^−1^, respectively). Conversely, the O–H stretching vibrations at 3288 cm^−1^ (Figure S7a) shift to lower frequencies, enhancing the hypothesis of new hydrogen-bond formation between cellulose and the polymeric matrix, as also discussed in previous works [17,18,39]. This observed shift indicates that both primary and secondary alcohol groups of cellulose are involved in forming hydrogen bonds with the polymer matrix. These interactions may also lead to conformational changes in the cellulose polymer chain, which could explain the marked reduction in intensity of the cellulose-associated bands at 985 and 1007 cm^−1^ in the composite spectra (indicative peaks are highlighted in Figure 4a).

Regarding the cellulose structure in the corresponding NLC-containing systems (Figure 4d–f), a similar discussion can be made as in the case of CNC. The contribution of peak intensity related to different conformations of primary and secondary alcohols appears to differ between NLC and CNC. Additionally, the frequencies of the characteristic C–O stretching vibrations of primary and secondary alcohols in the NLC spectrum show slight differences compared to CNC (1031 and 1055 cm^−1^). Within the composites, shifts are observed only for the secondary alcohol vibration (from 1055 to 1059 cm^−1^), suggesting that alterations occur mainly in these groups. It should be noted that in the high-frequency spectral window, due to spectral complexity in the region, potential shifts of the O–H stretching vibrations are not clearly discernible (Figure S7b).

According to the above, two observations warrant further investigation. First, in the chitosan/nanocellulose composites (Figure S6), the imprint of cellulosic inclusion is not distinct in the spectrum of the respective composites. Chitosan and cellulose, both being polysaccharides with similar 1–4 linked glycosidic structures, show similar spectral contributions especially in the 800–1200 cm^−1^ spectral window, with amorphous chitosan exhibiting broader peaks. As a result, any discrete cellulose peaks become spectrally buried under the strong chitosan absorptions, rendering them invisible in the composite spectrum, particularly considering the relatively low loading ratios of cellulose material (less than 10% and as low as 1%) [40]. Second, chitosan chains form an extensive intra- and intermolecular hydrogen bond network (involving NH_3_^+^ and C-OH groups) [41], which competes with potential cellulose–chitosan interactions.

The limited (to non-observed, in the case of CNC) spectral shift or intensity of the protonated amine str. vibration at 1545 cm^−1^ (Figure S6) probably highlights that chitosan–cellulose interactions are not significantly important. Apart from the changes corresponding to those of PVA composites in the C–O vibrations of cellulose, the simultaneous shift of the vibration of the protonated amino group (in any case of ternary systems) can account for the enhanced interaction of chitosan with PVA. The already known changes caused by the presence of cellulose in the PVA –OH environment likely allow oxygen atoms to act as hydrogen bond acceptors, with the protonated amino group of chitosan acting as the donor.

Summarizing these findings, in the ternary chitosan/PVA/nanocellulose composites, two distinct interactions were identified by ATR-FTIR spectroscopy. First, in chitosan–cellulose binary blends, the spectra remained essentially unchanged relative to neat chitosan, indicating that the chitosan–chitosan interactions dominate over the chitosan–cellulose ones. This is further confirmed in the hybrid composites by the absence of any appreciable shift in the protonated-amine deformation band at ~1550 cm^−1^ upon cellulose addition, signifying that the intrinsic chitosan network is more affected by PVA blending ratio. Second, cellulose–PVA interactions were evidenced by consistent shifts of the C-O stretching vibrations of cellulose (originally at ~1030 and ~1050 cm^−1^ shifted by ~4 cm^−1^) in both the binary PVA–cellulose and ternary systems. Thus, while cellulose remains spectrally absent within the chitosan domains, its hydroxyl groups continue to engage PVA chains via hydrogen bonding, enhancing the affinity of chitosan with PVA and improving miscibility at any blending ratio.

##### Study of the Effect in Crystal Structure

Chitosan is a polysaccharide whose native crystalline polymorphs arise from ordered hydrogen-bond networks between adjacent glucosamine chains. According to [42], four distinct chitosan conformations have been identified, one native crystal form and three acid-salt derivatives, all adopting variants of the extended two-fold helix. However, commercial medium molecular weight chitosan (e.g., Sigma-Aldrich, 75–85 % deacetylated) is typically isolated in a largely amorphous state due to its variable degree of deacetylation, chain length heterogeneity, and residual content. Consequently, its X-ray diffraction pattern is dominated by a broad amorphous halo centered around 20°, reflecting the lack of long-range order. Nevertheless, upon drying or mild annealing, intermolecular hydrogen bonds are reorganized to produce a partially ordered polymorph, which manifests as a weak diffraction peak at approximately 12°. This feature is attributed to the regular spacing of chitosan chains bridged by interchain hydrogen bonds and bound water molecules and serves as a spectroscopic indicator of formed “crystalline” domains within an otherwise amorphous matrix [43].

**Figure 5:**
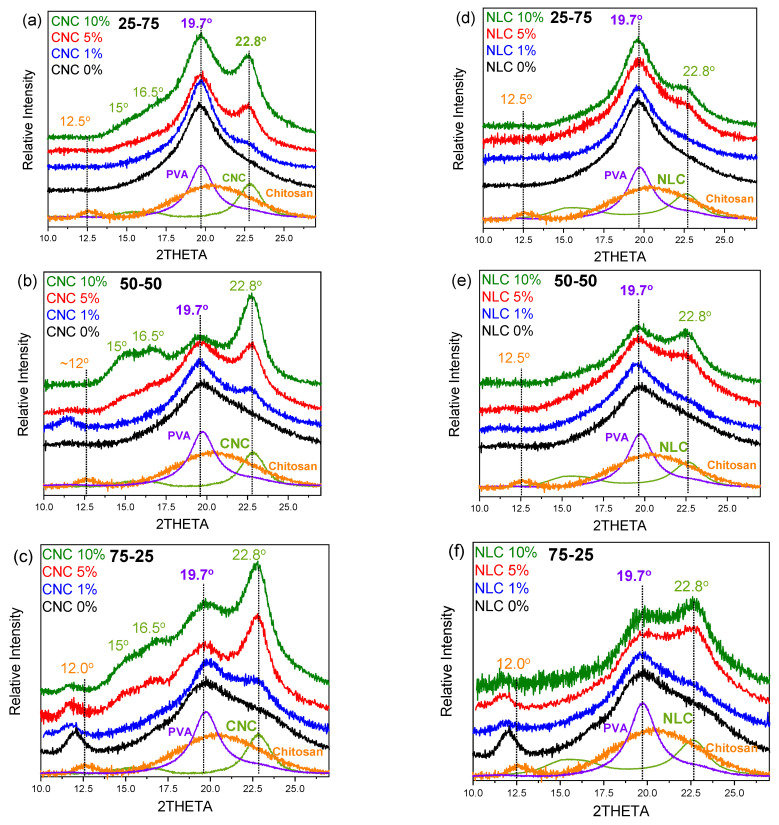
X-ray diffraction graphs of chitosan/PVA/nanocellulose composites: (**a**) 25-75 x% CNC, (**b**) 50-50 x% CNC, (**c**) 75-25 x% CNC, (**d**) 25-75 x% NLC, (**e**) 50-50 x% NLC, (**f**) 75-25 x% NLC.

Figure 5a shows the X-ray diffraction graphs of both initial cellulosic materials, chitosan/PVA matrix, and relevant blend/x% nanocellulose composite series. Reference nanocellulose materials (CNC and NLC) possess a characteristic sharp peak at 22.7° (200) and a doublet of peaks at ~15° ($1\bar{1}0$) and ~16.5° (110) assigned to the cellulose I_β_ allomorph [44].

As previously discussed, in the binary systems, the primary diffraction peak of PVA at 19.7° remains unaffected, while chitosan contributes both to the amorphous phase, as evidenced by a broad peak at around 20° and the characteristic peak at ~12°, which is associated with regions of partial crystalline organization. Upon incorporation of CNC (Figure 5a–c), the sharp diffraction peak of cellulose at 22.8° emerges, with its intensity increasing monotonically from 1% to 10% loading, while a similar increment is observed at the other CNC peaks too. In contrast, the NLC composites (Figure 5d–f) display lower contribution of cellulose (22.8°) within the broad blend’s diffraction profile, even at 10 wt% loading. This sparse NLC diffraction arises from its lower intrinsic crystallinity and from the micrometer-length fibers’ tendency to lie in different order than CNC, to the film plane, thereby reducing the population of lattice planes favorably oriented for effective diffraction. Consequently, despite equivalent mass fractions, CNC is far more “traceable” than NLC, highlighting the influence of filler crystallinity, size, and orientation on the detectability of nanofillers in polymer composites.

In any case, no differences in peak position or other diffraction profile arises, indicating that the crystalline structure of chitosan/PVA blend is largely maintained after the incorporation of either CNC or NLC. As the applied conditions were mild and only the minimum sufficient conditions to ensure solubility and film formation, by employing only a mild acid concentration and casting at room temperature to dissolve chitosan and form films without inducing thermal crystallization, each component shows its own crystal structure contribution in the blend graphs, suggesting that no crystallization of chitosan or other changes were achieved in any crystal structure. The fact that no shifts in chitosan, PVA, or cellulose contribution can be observed indicates that the incorporation of both nanocelluloses does not affect the structural uniformity of the polymer blend matrix but rather improves molecular ordering in the amorphous regions.

Finally, crystallinity index calculations of the PVA contribution within the composite become challenging and inaccurate due to overlapping peaks and baseline complexity within the area of interest. In contrast, DSC, where the PVA melting endotherm appears as a distinct peak, offers a more reliable and precise means of quantifying the crystalline content in these composites.

**Figure 6:**
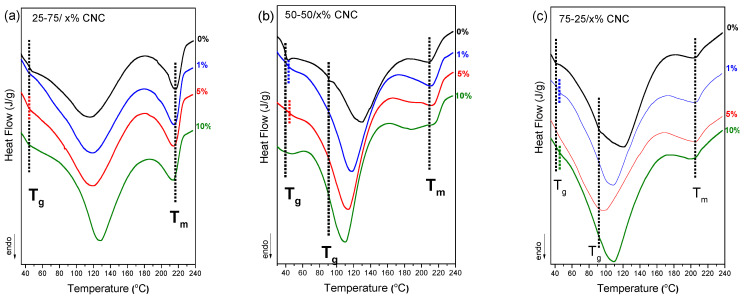

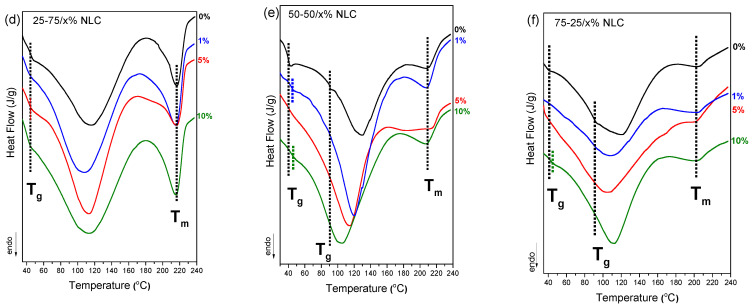
DSC thermographs of chitosan/PVA/nanocellulose composites: (**a**) 25-75 x% CNC, (**b**) 50-50 x% CNC, (**c**) 75-25 x% CNC, (**d**) 25-75 x% NLC, (**e**) 50-50 x% NLC, (**f**) 75-25 x% NLC.

Differential scanning calorimetry (DSC) analysis was performed to investigate the thermal behavior and phase compatibility of chitosan/PVA nanocellulose composites with varying CNC and NLC content, as depicted in Figure 6. In the control samples (0% nanocellulose), the 50-50 and 75-25 chitosan/PVA blends show limited miscibility as discussed earlier, with two distinct T_g_ values being observed in both cases. Upon incorporation of CNC at 1–10% concentrations, a single T_g_ was observed across all compositions (Figure 6a–c). The appearance of a single, well-defined glass transition temperature (T_g_) indicates the formation of a more homogeneous polymer network and serves as evidence of enhanced miscibility. Nanocellulose, due to its ability to form strong hydrogen bonds with the hydroxyl groups of PVA and, in turn, PVA with the amino groups of chitosan acts as a compatibilizer by reinforcing intermolecular interactions. This behavior likely explains the observed improvement in polymer miscibility across all blend ratios. Similar results have been observed in the case of the NLC composite blends (Figure 6d–f).

Moreover, the degree of crystallinity of the PVA phase in each chitosan/PVA/nanocellulose composite was quantified by normalizing the enthalpy of the endothermic melting peak to the PVA mass fraction. As summarized in Table 3, the 1% nanocellulose inclusions consistently yielded the highest crystallinity indices across all blend ratios, reaching approximately 40-45 % for 25-75 composites (40.3% CNC, 46.7% NLC), ~37 % for 50-50 composites, and ~50 % for 75-25 based membranes (55.0% CNC, 48.0% NLC). In each series, further increases in filler content (5 % and 10 %) led to a gradual reduction in PVA crystallinity, suggesting that low levels of nanocellulose act as more effective nucleating agents, whereas for higher loadings, despite also enhancing crystallinity, the increment is lower [45,46]. Furthermore, the melting temperature (T_m_) was consistently observed to be almost the same value after each nanocellulose inclusion, apart from 50-50 blend, in which CNC, in particular, expands the melting endotherm peak.

#### 3.2.2. Macroscopic Properties

##### Gas Permeability Measurements

In contrast to the already studied oxygen and water vapor permeability in chitosan/PVA and chitosan/PVA/cellulose composites intended for food packaging materials [24,26,47], carbon dioxide permeability remains largely uncharted to date. Since CO_2_ plays a pivotal role in several packaging applications (some of them requiring blockage and others requiring controlled permeation), where precise control of the gas composition is required to suppress microbial growth, retard enzymatic browning, and extend the shelf life of fresh product, relevant CO_2_ permeability measurements are considere a necessity [48,49]. Because CO_2_ exhibits a higher solubility and distinct quadrupolar interactions with polymer chains compared to O_2_ and N_2_, its diffusion mechanism and barrier requirements diverge significantly from those of other gases.

**Figure 7:**
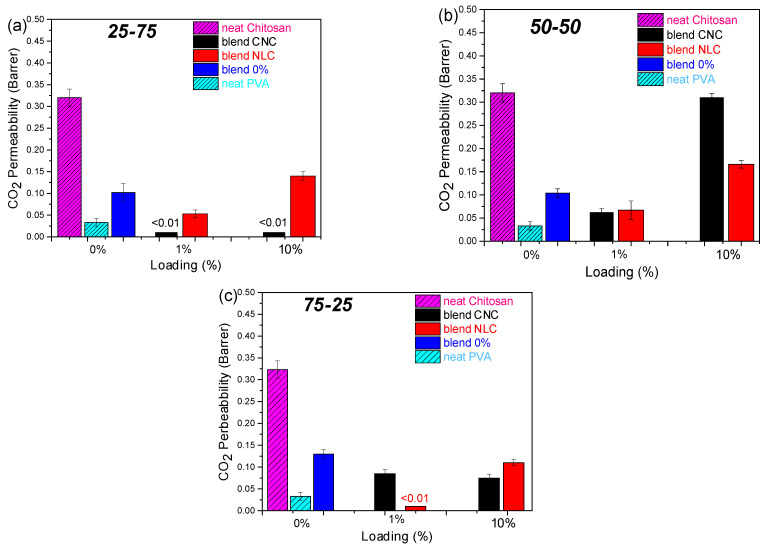
CO_2_ permeability (Barrer) bar plot versus cellulose loading of chitosan/PVA/nanocellulose composites: (**a**) 25-75 (**b**) 50-50, and (**c**) 75-25. Values < 0.01 Barrer are lower the detection limit.

Gas permeability measurements were performed on all chitosan/PVA/nanocellulose composites to elucidate the influence of filler type, loading level, and polymer blend ratios on CO_2_, O_2_, and N_2_ permeability properties for potential packaging applications (Figure 7). Neat chitosan (Figure S8) exhibited the highest CO_2_ permeability (0.32 Barrer), while neat PVA displayed the lowest value (0.033 Barrer); the neat 25-75, 50-50, and 75-25 blend membranes yielded intermediate permeabilities from 0.12 to 0.20 Barrer.

A pronounced suppression of CO_2_ permeability was observed at 1 wt% loading for both CNC and NLC across all matrix compositions, in agreement with the maximization of crystallinity index determined by DSC. At this low filler concentration, CNC and NLC act as efficient nucleating agents, promoting denser crystalline regions within the PVA phase and thereby reducing the amorphous free volume available for gas diffusion [50]. Optimal dispersion further extends the diffusion pathway, enhancing tortuosity and barrier performance. By contrast, higher filler loadings induce particle aggregation and localized micro-void formation, which compromise matrix consistency and elevate gas permeability [51].

However, apart from these general observations on wt% loading effect, incorporation of CNC into the PVA-rich matrix (25-75) shows a dramatic permeability reduction at 1 wt% CNC, where permeability fell below the detection limit (<0.01 Barrer) and remained suppressed even at loadings up to 10%. In contrast, NLC showed a preferential behavior to the chitosan-rich blend (75-25), in which total blockage of CO_2_ was observed. Both nanocellulose types at 1% loading on the 50-50 blend showed an equivalent 50% reduction in permeability, while higher loading in this case further disrupting the matrix consistency led to high CO_2_ permeation. Furthermore, neat PVA exhibited minimal O_2_ and N_2_ permeation (0.0023 and 0.0013 Barrer, respectively), which was entirely suppressed upon incorporation of either nanocellulose. These selective enhancements of the CO_2_ barrier properties are ascribed to preferential interactions and the network formation of CNC within PVA domains [17], as well as long-fiber NLC within chitosan domains, both of which increase path tortuosity and diminish the free volume in the corresponding matrices.

##### Mechanical Properties

**Figure 8:**
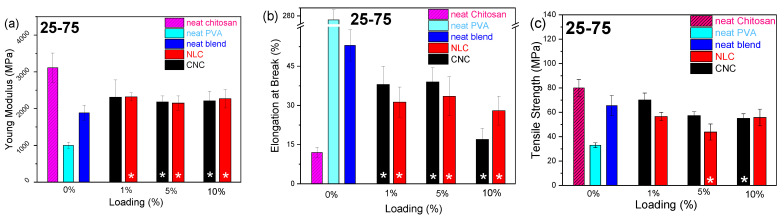

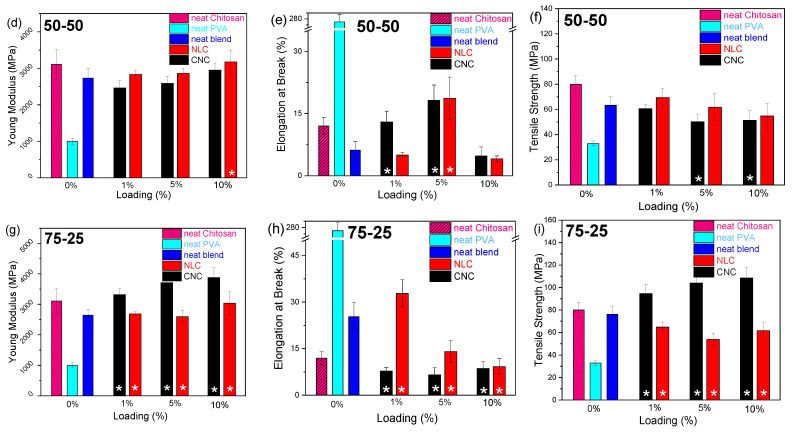
Young’s modulus (Y.M), elongation at break (E.B) and tensile strength (T.S) bar plots vs. loading of chitosan/PVA/nanocellulose composites. CNC composites (black), NLC composites (red), and neat blends (blue): (**a**) 25-75 Y.M, (**b**) 25-75 E.B, (**c**) 25-75 T.S. (**d**) 50-50 Y.M, (**e**) 50-50 E.B, (**f**) 50-50 T.S. (**g**) 75-25 Y.M, (**h**) 75-25 E.B, (**i**) 75-25 T.S. Asterisks (*) indicate level of statistical significance *p* ≤ 0.05.

A comparative evaluation of the mechanical properties is essential when introducing a multiparametric composite system for food packaging applications. The tensile strength, Young’s modulus, and elongation at break have been quantified across the varying polymer blend ratios and nanocellulose types/loadings with rigid CNC versus flexible, with the extended NLC fibrous network offering different contributions. Figure 8 shows a summarizing map of the Young’s modulus, elongation at break, and tensile strength bar plots versus loading, with all the data collected from typical stress–strain curves (25-75 in Figure S10, 50-50 in Figure S11, and 75-25 in Figure S12).

The reinforcing effect of CNC on the chitosan/PVA blends was first examined (Figure 8, black bars). In PVA-rich matrices (25-75 chitosan/PVA, Figure 8a–c), CNC addition induced a modest increase in Young’s modulus from 1.8 to 2.3 GPa, while the elongation at break was suppressed at moderate loadings (1–5%), and the tensile strength declined from ~65 to ~45 MPa. However, the presence of nanocellulose led to a slight decreasing trend in ductility across all loading levels, suggesting that the presence of nanocrystals hinders the effective transfer of mechanical stress between polymer chains [52]. This behavior contrasts with previous reports, in which chitosan/PVA/nanocellulose composites containing low chitosan content (<30 wt%) exhibited a slight enhancement in ductility upon the addition of small amounts of nanocellulose [22,23,47]. In the 50-50 system (Figure 8d–f), the addition of CNC influenced both the Young modulus and tensile strength in a manner similar to the PVA-rich blend. The presence of CNC, previously shown to act as a compatibilizing agent between PVA and chitosan by enhancing their miscibility, also facilitated stress transfer between polymer chains due to its high specific surface area and uniform dispersion. This resulted in a substantial improvement in the composite’s ductility (~70% increase at 5 wt% CNC loading). Notably, the dispersion quality played a crucial role, as evidenced by the 10 wt% CNC composites, where poor dispersion led to a markedly reduced tensile strength. Conversely, in chitosan-rich blends (75-25, Figure 8g–i), CNC incorporation yielded the most pronounced modulus and tensile strength gains (up to 25% at 10% loading), with the elongation at break being significantly suppressed (~80%), similarly to chitosan composites (Figure S8).

The impact of long-fiber NLC (Figure 8, red bars) differed markedly only in some cases. In the 25-75 blend, the lignocellulose increased the modulus similarly to CNC at equivalent loadings, despite the 250% increase noted in neat PVA composites, which is probably due to different relevance with the newly formed hybrid chitosan–PVA matrix [17]. The negative effect of NLC on matrix ductility remained similar to the PVA, reaching an almost 100% decrease in higher loadings. In the 50-50 composites, NLC enhanced the elongation, maximized at 5 wt%, as with CNC, while maintaining elasticity and tensile strengths comparable to the unfilled blend, indicating effective stress transfer without embrittlement, again enhancing the compatibility of both polymers. In the 75-25 chitosan/PVA system, the incorporation of 1 wt% NLC yielded a marked increase in the elongation at break (approximately from 20 % to 35 %), while the Young’s modulus remained essentially unchanged across all loadings. At low to moderate loadings, the inherently high stiffness of the rigid NLC fibers contributed to the composite modulus in a manner similar to that of the chitosan/PVA matrix, yielding negligible net change in initial elastic response. The ultimate tensile strength, by contrast, is governed by the efficiency of stress transfer at the fiber–matrix interface, which may be compromised by the formation of agglomerated domains that act as stress concentrators.

Table S1 summarizes the mechanical properties of the chitosan/PVA composite membranes, including the Young’s modulus, elongation at break, and tensile strength values. The results are reported as mean values ± standard deviation (SD) based on five independent measurements (*n* = 5). Statistical analysis was performed to assess differences among groups, with variations considered significant at *p* < 0.05.

##### Overall Comparison and Selection of the Appropriate Composites

**Figure 9:**
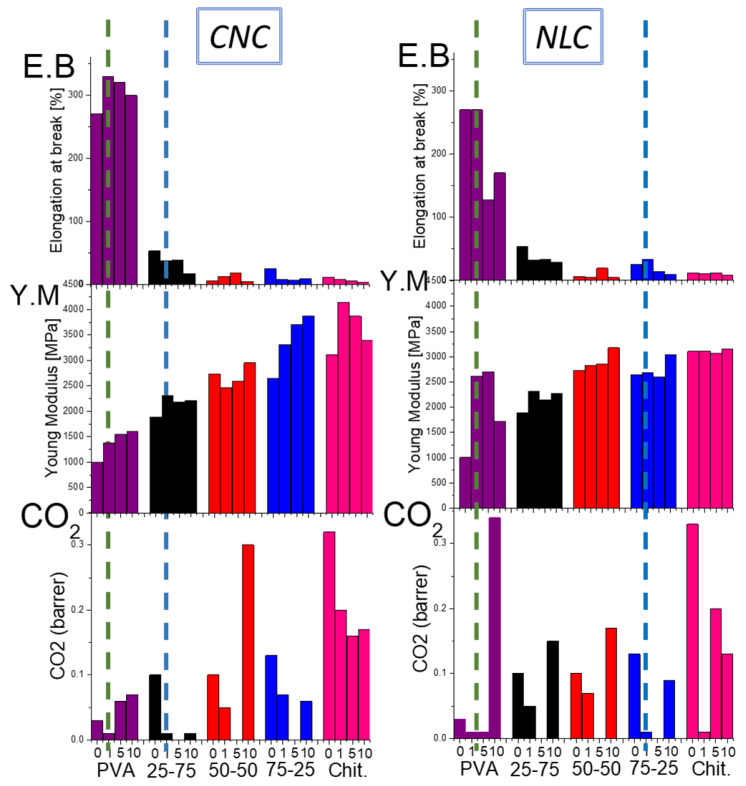
Comparative chart of the main functional properties (elongation at break, E.B, modulus of elasticity, Y.M, and CO_2_ permeability) of all possible combinations of CNC and NLC composites, aiming at the selection of the appropriate composites for each application.

In an effort to provide an overall comparison and selection of the appropriate composites for packaging applications with specific property requirements, comparative charts of the most useful functional properties, such as ductility (E.B), modulus of elasticity (Y.M), and CO_2_ barrier, are presented (Figure 9).

The matrix-selective reinforcement and CO_2_ barrier enhancements offered by CNC and NLC, then, can be understood in terms of filler/polymer affinity, dispersion quality, and network formation, which together govern both the mechanical and permeability properties. CNC, with its highly hydroxylated surface and nanoscopic dimensions, exhibits superior compatibility and uniform dispersion within PVA-rich domains with the preferable interaction promoting crystalline PVA nucleation, yielding CO_2_ tortuosity. Conversely, long-fiber NLC preferentially interacts with chitosan via entanglement with the polysaccharide backbone, which maximizes CO_2_ pathway tortuosity, while the network of NLC interrupts the matrix affinity due to enhanced formation of agglomerating domains.

Through this study, one can easily identify the optimal composite according to the property demands of the preferred application. For example, if the application requires packaging with high ductility while being a gas barrier (as in a modified atmosphere packaging, MAP), then PVA composites with CNC or NLC at a 1% concentration are suitable (Figure 9 green dot lines), whereas if the preferred property is an increased modulus of elasticity (as in a rigid packaging), the chitosan-rich 75-25 blend with 1% NLC and the 25-75 blend with 1% CNC are ideal (Figure 9 blue dot lines). The selective enhancement of mechanical properties and CO_2_ barrier properties with the introduction of CNC and NLC can be understood by studying the relevance of each inclusion with the two polymeric matrices and the quality of their dispersion, which together determine the functional properties of the composites. The preferable compatibility, improved molecular orientation and uniform dispersion in the case of PVA is achieved by introducing CNC, which brings about the presence of active crystallization centers of PVA with their beneficial effect regarding the functional properties of the composites [17]. Corresponding observations were made in this work in the case of composites rich in PVA (25-75). On the contrary, lignocellulose shows optimal affinity with chitosan (and mixtures rich in chitosan), which at 1% loading maximizes the labyrinth network without observing chitosan crystallization, improving the barrier to CO_2_ while not affecting the mechanical properties of the composites. However, its aggregation at higher concentrations negatively affects all their functional properties.

## 4. Conclusions

This study has highlighted the key characteristics and critical parameters that determine the functionality of chitosan/PVA/nanocellulose composite membranes for potential food packaging applications. Firstly, it was confirmed that the chitosan/PVA ratio is the primary factor governing their miscibility. The 25-75 (chitosan/PVA) blend exhibited a single-phase morphology, a smooth surface (as observed via SEM on cross-sectional images), and thermal behavior indicative of a homogeneous phase (i.e., a single intermediate glass transition temperature, T_g_). In contrast, the 50-50 blend presented phase separation, negatively impacting mechanical properties. This was supported by the appearance of structural discontinuities (observed voids in SEM images) and the presence of two distinct T_g_ values, with each one corresponding to each polymer. The 75-25 composition showed intermediate miscibility characteristics.

The incorporation of nanocellulose, either as cellulose nanocrystals (CNC) or nano-lignocellulose (NLC), proved to play a catalytic role in developing the crystalline network of PVA, particularly at 1 wt% loading. At this concentration, the highest crystallinity values were recorded for all blend ratios (~40–55%), which can be attributed to the nucleating action of nanocellulose. Moreover, optimal dispersion at low loadings resulted in remarkably low CO_2_ permeability values (<0.01 Barrer) for CNC composites at the 25-75 ratio, with similarly low values observed for NLC composites at the 75-25 ratio (chitosan/PVA). In contrast, higher loadings (5–10 wt%) negatively affected performance, reducing PVA crystallinity and significantly increasing CO_2_ permeability. This is likely due to filler agglomeration and the presence of localized areas within the matrix devoid of filler/nucleation sites.

The choice of nanocellulose type showed distinct effects depending on the polymer matrix: CNC was more effective in PVA-rich blends, enhancing CO_2_ barrier performance and elasticity, while NLC improved the performance of chitosan-rich composites by forming a more tortuous diffusion path and thus minimizing gas permeation across the membrane. SEM and ATR-FTIR analyses confirmed that CNCs, due to their ideal aspect ratio and uniform dispersion, formed a stable network within the PVA matrix through extensive hydrogen bonding. In contrast, NLC, due to its longer fibers and weak (or absent) interaction with chitosan, was prone to aggregation even at low concentrations.

Mechanical testing revealed that the addition of 1 wt% CNC in the 25-75 composites increased both the tensile strength and Young’s modulus without significantly compromising ductility. Similarly, 1 wt% NLC in the 75-25 composites enhanced the tensile strength, with the highest ductility observed at low filler concentrations. At higher concentrations (5–10 wt%), a decline in coherence and a sharp reduction in ductility were observed due to agglomeration.

The observed hydrogen bonding network between PVA and cellulose within the ternary composites, combined with the chitosan–PVA interaction, underscores the dual role of nanocellulose: enhancing functional performance and simultaneously promoting miscibility between PVA and chitosan. As revealed by XRD, the incorporation of either type of nanocellulose does not disrupt the structural uniformity of the matrix but improves molecular ordering in the amorphous regions, as reflected in increased crystallinity (DSC) and significantly improved gas barrier properties.

Based on a detailed analysis of both mechanical and permeability properties, it was clearly demonstrated that chitosan/PVA/nanocellulose membranes can be tailored to deliver optimized gas barrier performance, enhanced mechanical strength, and elasticity while minimizing environmental impact due to the use of biodegradable, edible raw materials. The performance of nanocomposites is directly influenced by the chitosan/PVA ratio, the type of nanocellulose used, and its loading percentage, offering a wide parameter space for tuning properties to suit modified atmosphere or even rigid packaging applications in an environmentally sustainable manner.

## Figures and Tables

**Table 1:** Summary of all prepared samples with their polymer ratios and nanocellulose content.

Sample Code	Chitosan/PVA Ratio	Nanocellulose Type	Loading (wt%)
0-100 0%	0-100	-	0%
100-0 0%	100-0	-	0%
25-75 0%	25-75	-	0%
25-75 1% CNC/NLC	25-75	CNC/NLC	1%
25-75 5% CNC/NLC	25-75	CNC/NLC	5%
25-75 10% CNC/NLC	25-75	CNC/NLC	10%
50-50 0%	50-50	-	0%
50-50 1% CNC/NLC	50-50	CNC/NLC	1%
50-50 5% CNC/NLC	50-50	CNC/NLC	5%
50-50 10% CNC/NLC	50-50	CNC/NLC	10%
75-25 0%	75-25	-	0%
75-25 1% CNC/NLC	75-25	CNC/NLC	1%
75-25 5% CNC/NLC	75-25	CNC/NLC	5%
75-25 10% CNC/NLC	75-25	CNC/NLC	10%

**Table 2:** Glass transition and melting temperature of chitosan–PVA blend membranes.

Sample Description	T_g_ (°C)	T_m_ (°C)
0-100	~45	227.0
25-75	~50	216.7
50-50	~45	210.0
75-25	~45	205.0
100-0	~90	Non-Detected

**Table 3:** Crystallinity index and melting temperature of chitosan/PVA composite membranes.

Sample Description	Crystallinity Index (%)	T_m_ (°C)
0-100 0%	41	227
100-0 0%	Non-Detected	Non-Detected
25-75 0%	26.0	216.7
25-75 1% CNC/NLC	40.5/46.5	215.0/215.0
25-75 5% CNC/NLC	39.0/43.0	215.0/215.0
25-75 10% CNC/NLC	33.0/43.0	213.5/215.0
50-50 0%	27.0	210.0
50-50 1% CNC/NLC	38.5/36.5	210.0/207.5
50-50 5% CNC/NLC	31.0/30.5	210.0/205.0
50-50 10% CNC/NLC	28.5/32.0	210.0/207.0
75-25 0%	39.0	205.0
75-25 1% CNC/NLC	55.0/48.0	205.0/205.0
75-25 5% CNC/NLC	52.0/41.0	205.0/205.0
75-25 10% CNC/NLC	38.0/39.0	202.0/205.0

## Data Availability

The original contributions presented in this study are included in the article/Supplementary Materials. Further inquiries can be directed to the corresponding authors.

## Supplementary Materials

The following supporting information can be downloaded at https://www.mdpi.com/article/10.3390/polym17233122/s1. Figure S1: Comparative analysis of various chitosan/PVA blend ratios: (a) stress–strain curves, (b) Young’s modulus vs. PVA content bar plots, (c) CO_2_ permeability vs. PVA content bar plots, and (d) optical representation of blend films. Figure S2: High magnification cross-section surface SEM images of chitosan/CNC composites: (a) 0%, (b) 1%, (c) 5%, and (d) 10% loading. Figure S3: Cross-section surface SEM images of chitosan/PVA/NLC composites at high magnification: (a) 25-75 1%, (b) 25-75 5%, (c) 25-75 10%, (d) 50-50 1%, (e) 50-50 5%, (f) 50-50 10%, (g) 75-25 1%, (h) 75-25 5%, (i) 75-25 10%, (j) neat PVA (0-100). The scale bar in all images corresponds to 1 µm. Figure S4: High magnification cross-section surface SEM images of chitosan/NLC composites: (a) 1%, (b) 5%, and (c) 10% loading. Figure S5: Optical images of chitosan/PVA/nanocellulose composites. From left to right, (a) 0-1-5-10% CNC, (b) 1-5-10% NLC, and (c) 5% NLC composites of 25-75, 50-50, and 75-25 blends. Figure S6: ATR-FTIR spectra of pure chitosan film, cellulosic inclusion, and chitosan composites: (a) 1%, 5%, and 10% CNC; (b) 1%, 5%, and 10% NLC. Figure S7: ATR-FTIR spectra at 4000-2600 cm^−1^ spectral window of pure chitosan, pure PVA, cellulosic inclusion, and 25-75 blend composites: (a) 1%, 5%, and 10% CNC; (b) 1%, 5%, and 10% NLC. Figure S8: CO_2_ permeability (Barrer) bar plot versus cellulose loading of chitosan/nanocellulose composites: CNC (black) and NLC (red). Values < 0.01 Barrer are lower than the detection limit. Figure S9: Young’s modulus (a), elongation at break (b), and tensile strength (c) bar plots vs. loading of chitosan/nanocellulose composites. CNC composites (black), NLC composites (red), and pure chitosan (blue). Figure S10: Stress–strain curves of 25-75 (chitosan/PVA) CNC composites (a), and NLC composites (b). The linear region (inlet). Figure S11: Stress–strain curves of 50-50 (chitosan/PVA) CNC composites (a) and NLC composites (b). The linear region (inlet). Figure S12: Stress–strain curves of 75-25 (chitosan/PVA) CNC composites (a) and NLC composites (b). The linear region (inlet). Table S1. Young’s modulus, elongation at break, and tensile strength of chitosan/PVA composite membranes.
