# Supplementary material for: Nanocellulose Filled Bio-Based PVA/Chitosan Nanocomposites: Structure–Property Relationships Toward Advanced Food Packaging Films
Source: Polymers (Basel). 2025 Nov 24;17(23):3122. doi: 10.3390/polym17233122 (PMC12694045; doi:10.3390/polym17233122)
Supplement: Supplementary file 1 [file polymers-17-03122-s001.zip › polymers-3949643-supplementary.pdf]

Supplementary Information

Nanocellulose filled Bio-Based PVA/Chitosan Nanocomposites: Structure–Property Relationships toward Advanced Food Packaging Films

Konstantinos Papapetros[1,2*], Georgios N. Mathioudakis[1], Dionysios Vroulias [1], Nikolaos Koutroumanis [3], Amaia Soto Beobide[1], Olympia Kotrotsiou[4], Giannis Penloglou[4], Konstantinos S. Andrikopoulos[1,5] and George A. Voyiatzis[1*]

[1] Foundation for Research and Technology—Hellas (FORTH), Institute of Chemical Engineering Science (ICE-HT), Stadiou St., GR 265 04 Patras, Greece; kpapapetros@iceht.forth.gr (K.P.); gvog@iceht.foth.gr (G.A.V) mathioy@iceht.forth.gr (G.N.M.); dvroulias@iceht.forth.gr (D.V.); asoto@iceht.forth.gr (A.S.B.)

[2] Department of Chemical Engineering, University of Patras, GR 265 04 Patras, Greece

[3] Application Driven Research & Innovative Engineering (ADRINE), Patras Science Park, Stadiou Street, Platani, GR 265 04 Patras, Greece; nkoutrou@adrine.gr (N.K.)

[4] Centre for Research and Technology Hellas (CERTH), Chemical Process and Energy Resources Institute (CPERI), 6th km. Charilaou-Thermi Rd, Thermi, GR 570 01, Thessaloniki, Greece; okotrots@certh.gr (O.K.); penloglou@certh.gr (G.P.)

[5] Department of Physics, University of Patras, GR 265 04 Patras, Greece; kandriko@upatras.gr

*****Correspondence: gvog@iceht.forth.gr (G.A.V.); kpapapetros@iceht.forth.gr (K.P)

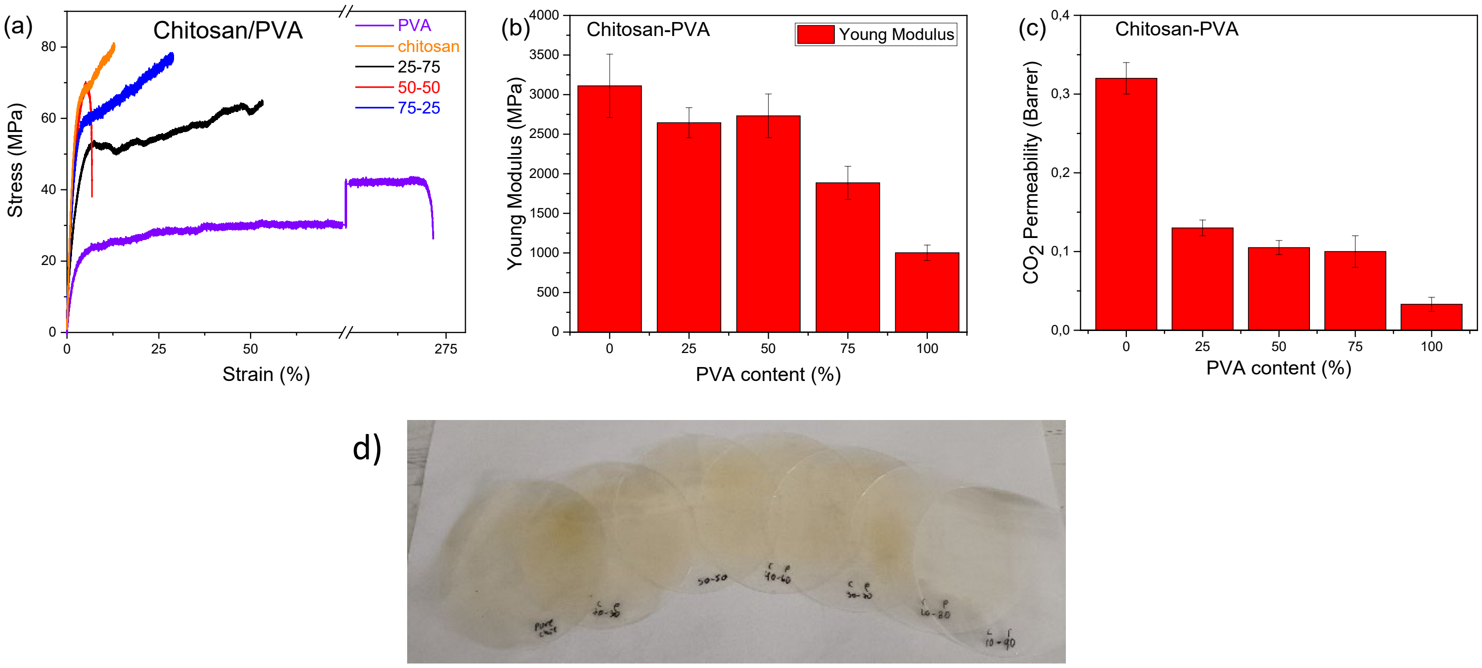

Figure S1. Comparative analysis of various Chitosan-PVA blend ratios: (a) stress-strain curves, (b) Young's Modulus vs PVA content bar plots, (c) CO_2 Permeability vs PVA content bar plots and (d) optical representation of blend films.

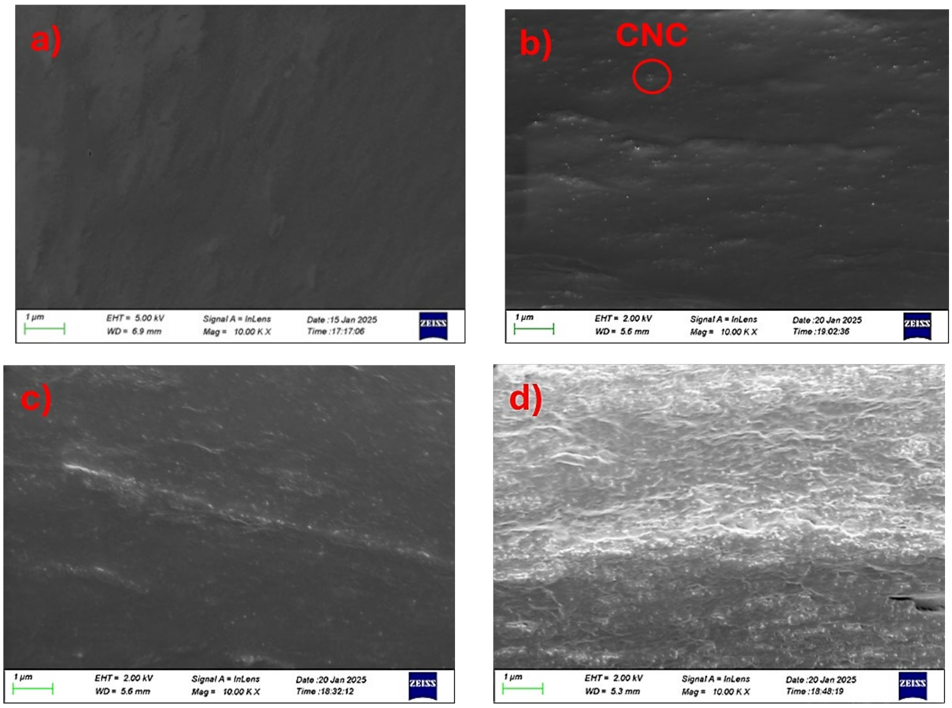

Figure S2. High magnification cross-section surface SEM images of Chitosan/CNC composites: (a) 0%, (b) 1%, (c) 5% and (d) 10% CNC loading. The scale bar in all images correspond to 1 µm.

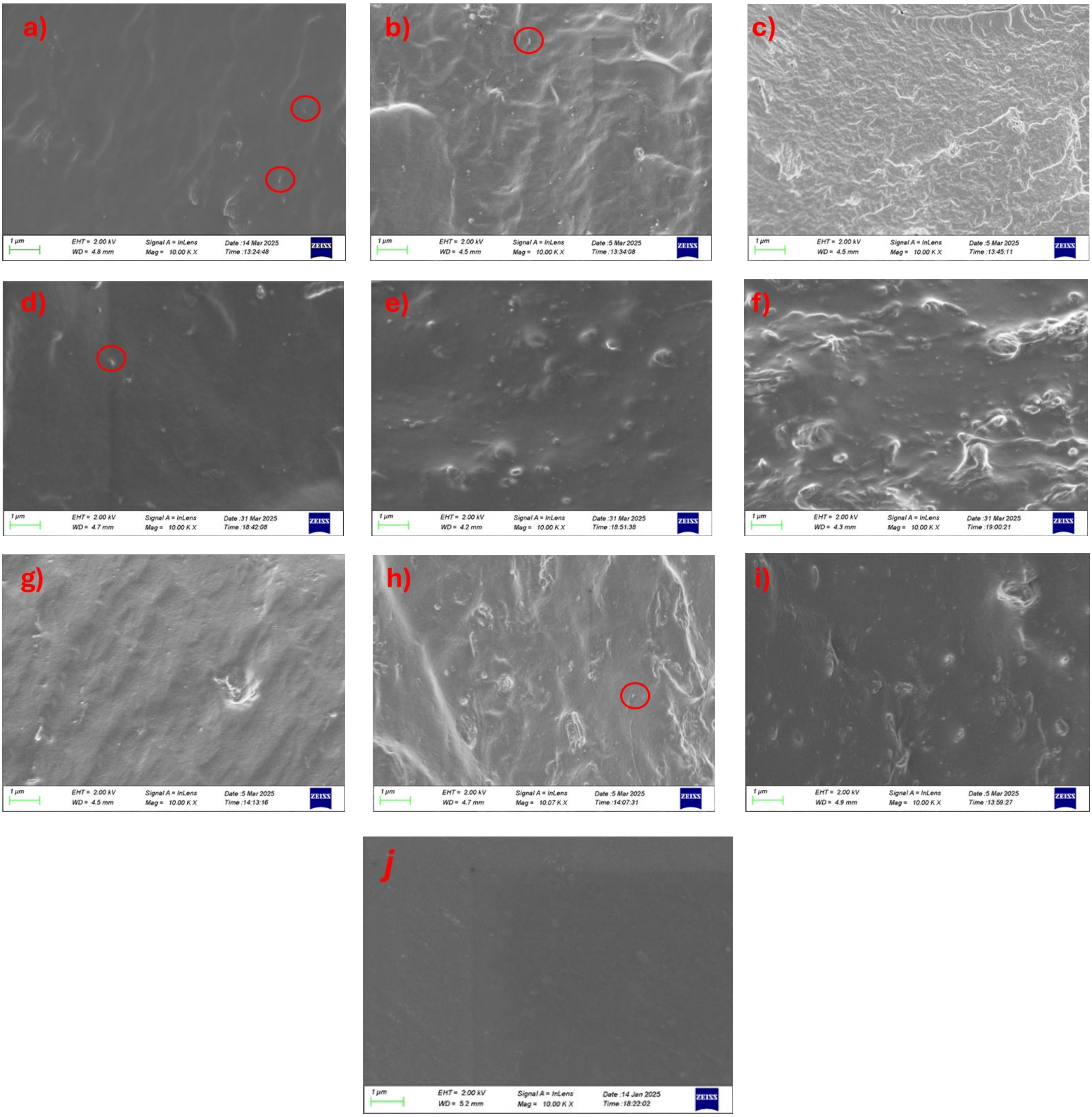

Figure S3. Cross-section surface SEM images of Chitosan-PVA/NLC composites at high magnification. (a) 25-75 1%, (b) 25-75 5%, (c) 25-75 10%, (d) 50-50 1%, (e) 50-50 5%, (f) 50-50 10%, (g) 75-25 1%, (h) 75-25 5%, (i) 75-25 10% and (j) neat PVA (0-100). The scale bar in all images correspond to 1 µm.

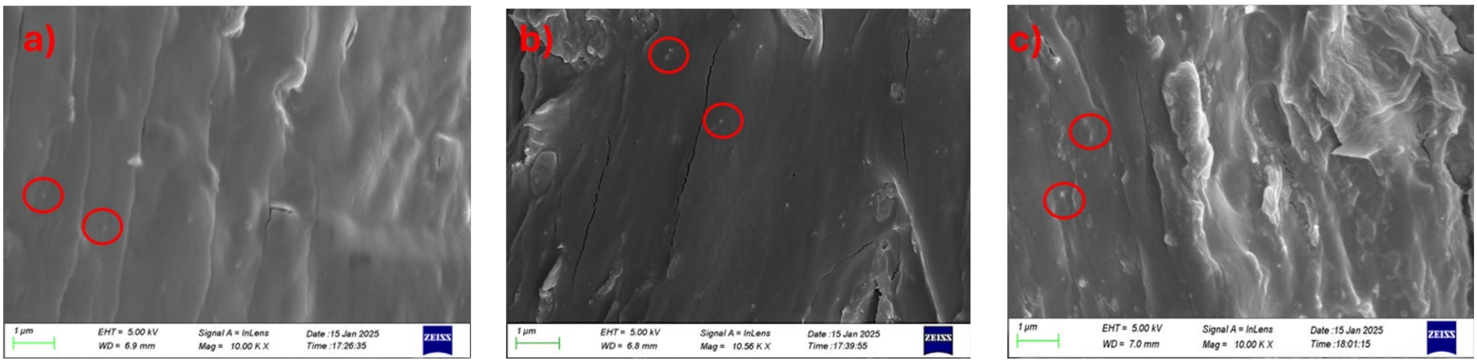

Figure S4. High magnification cross-section surface SEM images of Chitosan/NLC composites: (a) 1%, (b) 5% and (c) 10% NLC loading. The scale bar in all images correspond to 1 µm.

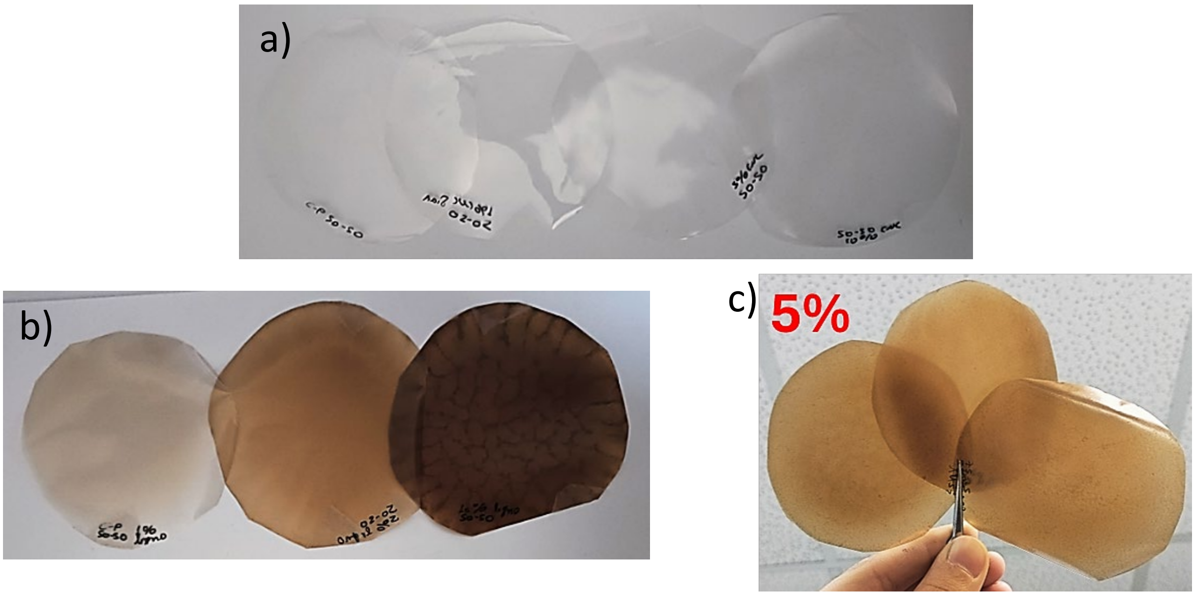

Figure S5. Optical images of Chitosan-PVA/nanocellulose composites, from left to right: (a) 0,1,5 and 10% CNC, (b) 1,5 and 10% NLC loading and (c) 25-75, 50-50 and 75-25 Chitosan-PVA composites containing 5% NLC loading.

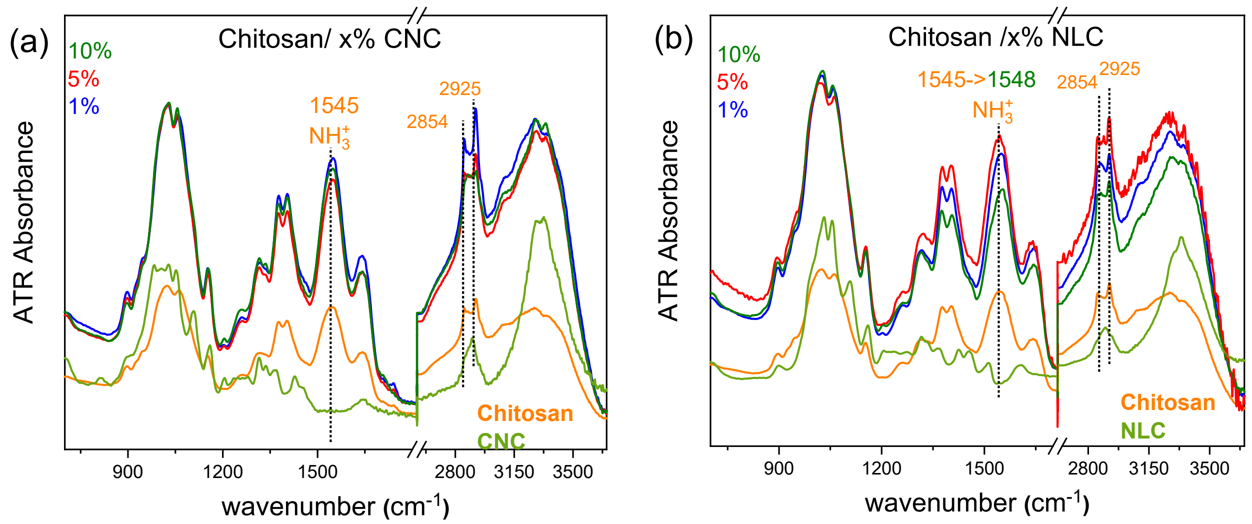

Figure S6. ATR/FTIR spectra of neat Chitosan film, cellulosic inclusion and Chitosan composites: (a) 1%, 5% and 10% CNC loading. (b) 1%, 5% and 10% NLC loading.

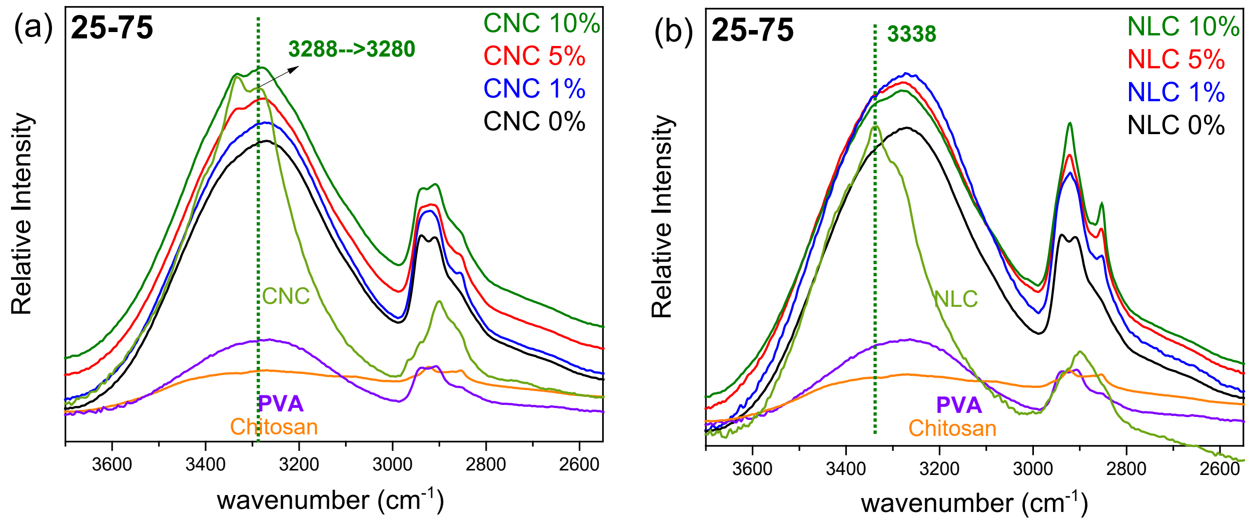

Figure S7. ATR/FTIR spectra at 4000-2600 cm^{-1} spectral window of neat Chitosan, neat PVA, cellulosic inclusion and 25-75 blend composites: (a) 1%, 5% and 10% CNC loading, (b) 1%, 5% and 10% NLC loading.

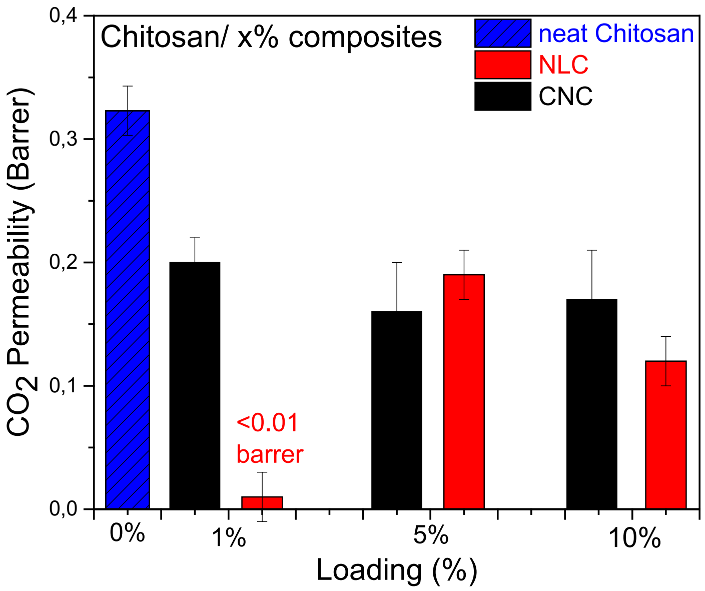

Figure S8. CO_2 permeability (Barrer) bar plot versus cellulose loading of Chitosan/nanocellulose composites: CNC (black) and NLC (red). Values <0.01 Barrer are lower than the detection limit.

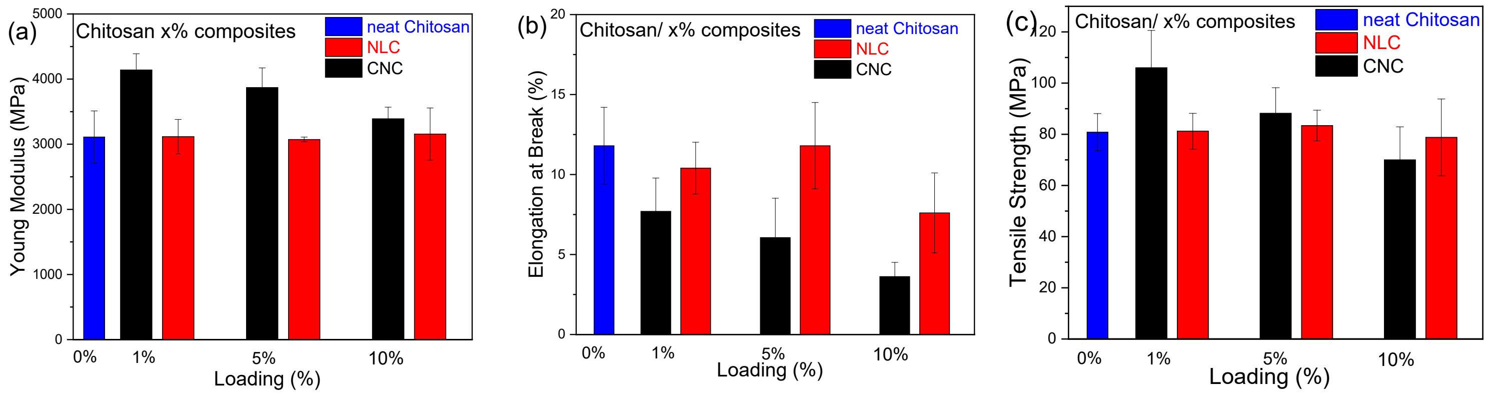

Figure S9. (a)Young Modulus, (b) Elongation at Break and (c) Tensile Strength bar plots vs cellulose loading of Chitosan/nanocellulose composites. CNC composites (black), NLC composites (red) and neat chitosan (blue).

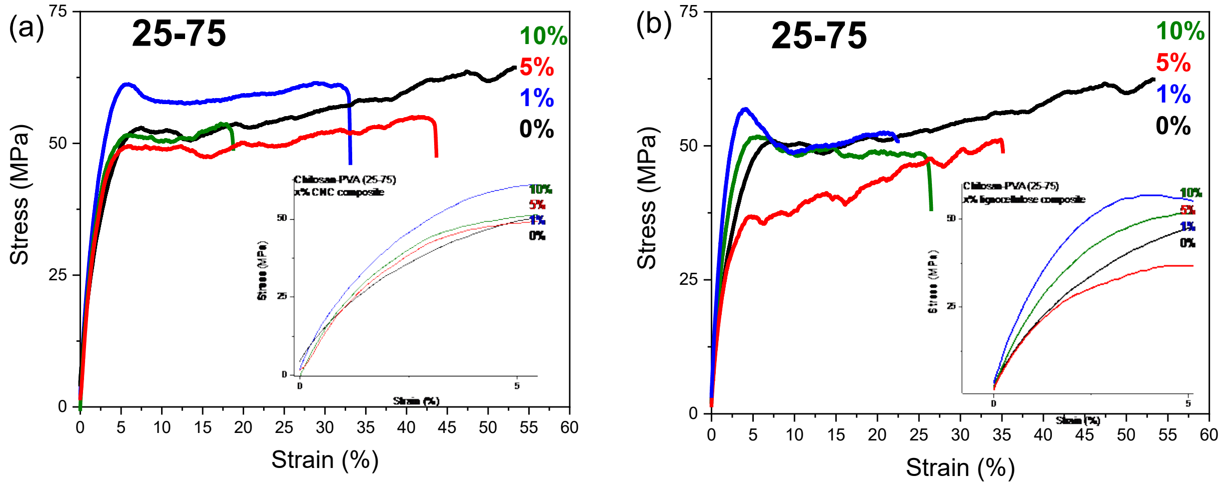

Figure S10. Stress-strain curves of 25-75 (Chitosan-PVA) (a) CNC composites and (b) NLC composites. The linear region (inlet).

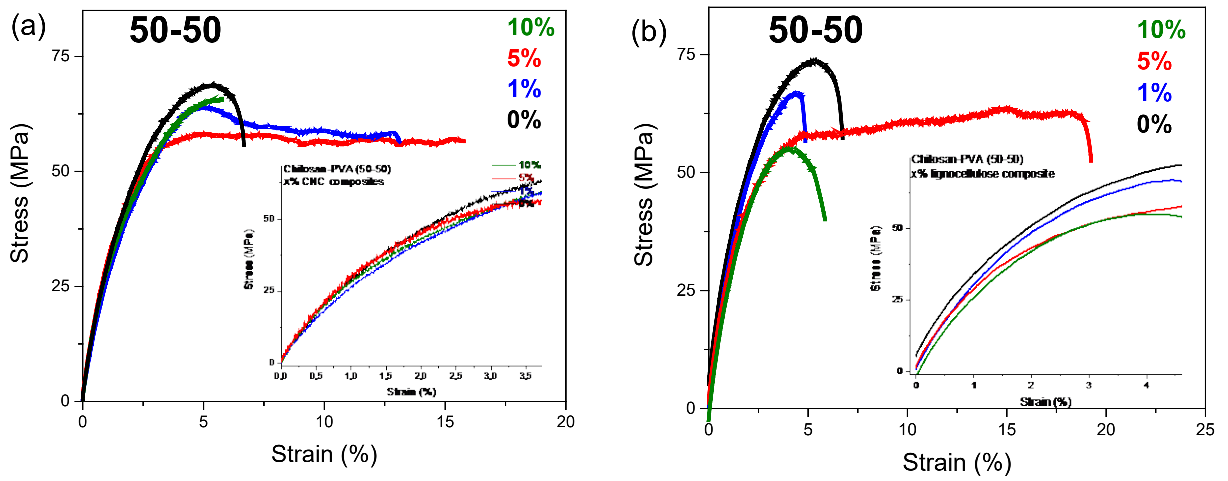

Figure S11. Stress-strain curves of 50-50 (Chitosan-PVA) (a) CNC composites and (b) NLC composites. The linear region (inlet).

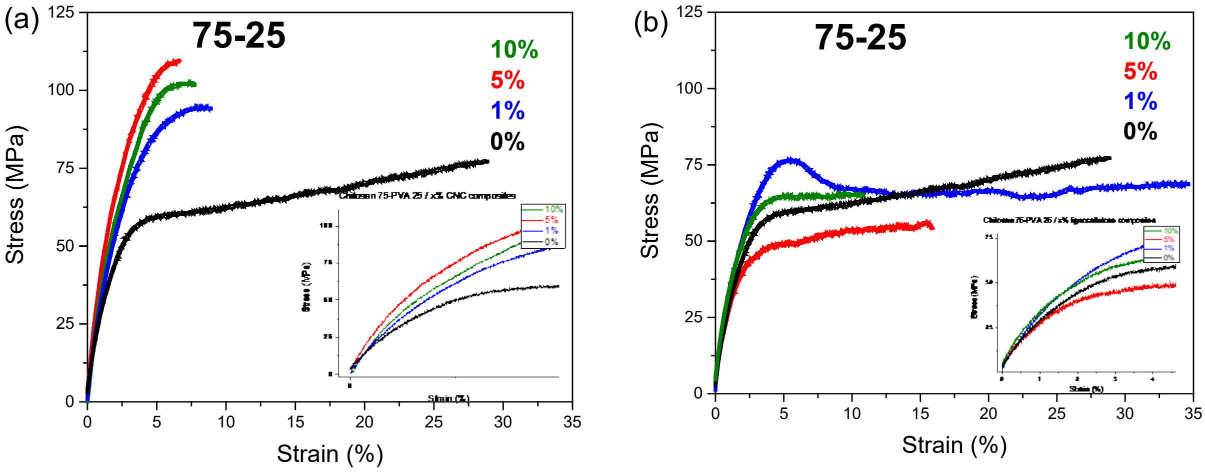

Figure S12. Stress-strain curves of 75-25 (Chitosan-PVA) (a) CNC composites and (b) NLC composites. The linear region (inlet).

Table S1. Young's Modulus, Elongation at break and Tensile Strength of chitosan/PVA composite membranes

Sample description	*Young Modulus (MPa)*	*Elongation at Break (%)*	*Tensile Strength (MPa)*
**0-100 0%**	1002±86	270±25	33±2
**100-0 0%**	3111±400	12±2	80±7
**25-75 0%**	1850±190	53±6	65±8
**25-75 1% CNC/ NLC**	2310±468 / 2320±108	38±7 / 31±6	70±6 / 56±3
**25-75 5% CNC/ NLC**	2180±167 / 2150±193	39±5 / 33±7	57±3 / 44±7
**25-75 10% CNC/ NLC**	2210±265 / 2270±250	17±4 / 28±5	55±4 / 56±7
**50-50 0%**	2732±276	6±2	63±7
**50-50 1% CNC/ NLC**	2462±198 / 2832±108	13±3 / 5±1	61±3 / 70±7
**50-50 5% CNC/ NLC**	2588±191 / 2861±139	18±4 / 19±5	50±5 / 62±11
**50-50 10% CNC/ NLC**	2950±180 / 3176±320	5±2 / 4±1	51±8 / 55±10
**75-25 0%**	1886±209	25±4	76±7
**75-25 1% CNC/ NLC**	3316±196 / 2686±77	8±1 / 33±4	95±8 / 65±4
**75-25 5% CNC/ NLC**	3710±229 / 2594±215	7±2 / 14±3	104±11 / 54±6
**75-25 10% CNC/ NLC**	3880±317 / 3037±385	9±2 / 9±2	109±9 / 62±7
